# Prevalence of substance use among Iranian male adolescents: Systematic review and meta‐analysis

**DOI:** 10.1002/hsr2.885

**Published:** 2022-10-25

**Authors:** Jalil Hosseini, Ehsan Shojaeefar, Parham Pooladgar, Fereshteh Aliakbari, Maryam Ganji, Mostafa Hamdieh, Ali Kheradmand, Mahta Abbasi Fashami

**Affiliations:** ^1^ Men's Health and Reproductive Health Research Center Shahid Beheshti University of Medical Sciences Tehran Iran; ^2^ Immunology Board for Transplantation and Advanced Cellular Therapeutics (ImmunoTACT) Universal Scientific and Education Network (USERN) Tehran Iran; ^3^ School of Medicine Shahid Beheshti University of Medical Science Tehran Iran; ^4^ Department of Psychiatry, Taleghani Hospital Research Development Committee, School of Medicine Shahid Beheshti University of Medical Sciences Tehran Iran; ^5^ School of Nursing and Midwifery Shahid Beheshti University of Medical Sciences Tehran Iran

**Keywords:** male adolescents, meta‐analysis, prevalence study, substance dependence

## Abstract

**Background and aims:**

Substance use among adolescents is one of the most challenging behavioral disorders with direct consequences. It is of the essence (that) the prevalence of substance use is investigated among Iranian male adolescents.

**Methods:**

The present study is a systematic review and meta‐analysis. All published articles titled “prevalence of substance use among Iranian adolescents” authored in Persian and English from 2004 to 2020 on Pub Med, Scopus, SID, and Google Scholar, a top list of academic research databases, were reviewed. Thirty‐three out of 805 articles, hinge on the inclusion and exclusion criteria, were eligible. Statistical analysis carried out in STATA 14.0.Q index, I2 index, and *χ* ^2^ test were applied.

**Results:**

Overall prevalence reported with (95% confidence interval) for substance use 7% (4%–11%), methamphetamine 4% (3%–6%), tobacco 10% (3%–19%), and any addictive substances 4% (2%–7%) among male adolescents respectively. Besides, overall prevalence of alcohol consumption reported 10% (8%–11%).

**Conclusion:**

The prevalence of substance use among male adolescents is high, so it is indispensable for researchers to pay special attention to this issue.

## 1 INTRODUCTION

Substance use always goes hand in hand with fundamental social and health problems. It is considered one of the major risk factors for other diseases, especially among adolescents.^1^, ^2^ substance use among adolescents is characterized by World Health Organization as one of the most serious behavioral challenges resulting in suicide, conflict, car accidents, high‐risk sexual behaviors plus dropping out of school, delinquency, the decline in social capital, and family breakdown as social obstacles and health issue such as infectious diseases (AIDS and hepatitis).^3^, ^4^, ^5^ In recent years, a myriad of large national studies indicated that popular and common substances in Iran are known as prevalent addictive substances used. Opium and Heroin are cases in point. Yet, substances use such as heroin, ecstasy, and stimulants are getting significantly increased.^6^, ^7^

According to the World Disease Burden Report, every year, the use of diverse substance categories among Iranian men accounts for 2.7% (2.29%–3.11%) of the years of life lost due to disability‐adjusted life years which is proximately 2.6 times more than are reported among Iranian women.^8^ Scattered reports of addictive substance use among Iranian adolescents illustrated that the prevalence of alcohol consumption is roughly 30% and tobacco and other substances are about 3%.^9^ It is supposed that adolescents, in particular, boys are more susceptible to have high‐risk behaviors and substance use than adults and girls might have, therefore it is needed to pay more attention and plan for gender and age categories.^10^ In the current systematic review and meta‐analysis, it is alleged that the prevalence of methamphetamine uses doubled among adolescent males compared to adolescent girls.^11^ This study is endeavored to help health policymakers to take preventive and serious risk reduction strategies and measures by clarifying the general situation of substance use among high‐risk gender and age groups. In this study, we decided to determine the prevalence of substance use among Iranian male adolescents.

## 2 MATERIALS AND METHODS

This study is a systematic review and meta‐analysis to estimate the prevalence of substance use among teenage boys during the last 15 years. Owing to confining this study to male adolescents, those who are substance users, it is needed to review more studies in a long duration (17 years). The further rationale is that the prevalence of synthetic substance use is getting bumped up in recent decades, particularly among Iranian populations and it might trigger complicated predicaments.^11^ To do so, all Iranian English and Persian articles entitled “the prevalence of substance use among adolescents” published on PubMed, Scopus, SID, and Google Scholar from 2004 to 2020 were collected and reviewed. Furthermore, papers presented at national seminars, congresses, national reports, and related dissertations were added to the collected database, and in case of being applicable, the full text of the articles was included. To find related articles on English academic research databases, the keywords “substance use,” “smoking,” “cigarette,” “tobacco use,” “hookah,” “water pipe,” “alcohol,” “heroin,” “hashish,” “cannabis,” “opium,” “methamphetamine,” “tramadol,” “cocaine,” “marijuana,” “morphine,” and “Iran,” linked with“ OR” and “AND” were selected based on the MESH.

**Figure 1:**
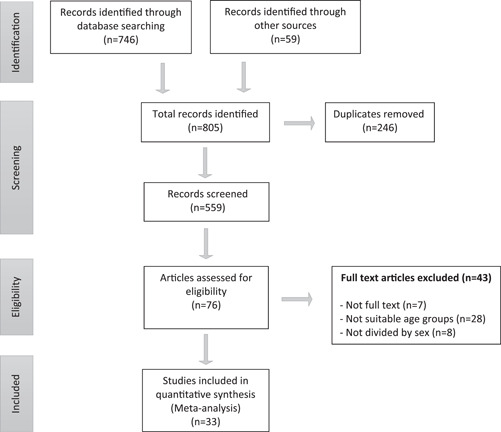
PRISMA flowchart of study selection

Study selection had been done according to PRISMA method as showed in Figure 1. First, 805 studies were extracted and relied on determinate keywords. After duplicate studies were eliminated in reference management software (endnote), the title and abstract of the studies were reviewed and evaluated by two independent researchers. In the next step, the remaining studies were evaluated completely and ultimate studies were selected for this study.

### 2.1 Exclusion criteria

- Studies have not reported the prevalence of substance use among male adolescents (10 to 24 years) in the recent 15 years.
- Not being possible to use primary data, review articles and letters to the editors were omitted.

To ensure that all studies are referable, a reference list of the final articles was also extracted.^4^, ^12^, ^13^, ^14^, ^15^, ^16^, ^17^, ^18^, ^19^, ^20^, ^21^, ^22^, ^23^, ^24^, ^25^, ^26^, ^27^, ^28^, ^29^, ^30^, ^31^, ^32^, ^33^, ^34^, ^35^, ^36^, ^37^, ^38^, ^39^, ^40^, ^41^, ^42^, ^43^

To extract data from the texts, two researchers explored the information independently using an unstructured form. Embodied items of the form are (1) Bibliographic information (first author, publication date, article ID, review date), (2) Type of study (descriptive, descriptive‐analytical, case‐control, cohort), (3) Study setting, and demographic information (total sample size and the number of male adolescents; age average, type of substance use (cigarettes, hookah, alcohol, etc.,) and history of use (never used, regular use, current use, and recreational use [experimenter]).

The quality of the studies was assessed using a valid and reliable instrument designed by Munn et al.^44^ in 2014 to measure the quality of the systematic review of prevalence studies. This instrument includes 10 questions measuring the quality of the method contains generalizability of samples, sample size, sampling, explanation of the subject characteristics, measurement method, data analysis, confounder control, and the general assessment of methodology. Answers were structured and scored by (Yes = 1, No, and Vague = 0). The range of scores was from 0 to 10 and studies with 5 and above were included in the study. This instrument ubiquitous and in several systematic reviews, studies applied worldwide to measure prevalence.^45^, ^46^, ^47^ As mentioned, quality assessment was performed by two authors independently and in case of disagreement, the third author would comment.

To analyze and integrate results, using the prevalence of substance use as an indicator, the standard error was obtained by the binomial distribution. In Stata (14.0, Metaprop command) combined prevalence was estimated from (either nearly 0 or about 100).^48^ Heterogeneity among results was assessed using the Q index and *χ* ^2^ test (*p* < 0.05) plus the I2 index.^49^ According to references, I2 > 50% indicates significant heterogeneity.^16^ The overall prevalence of substance use estimation was found by the random effect model applied by reversing the variance method. To reduce heterogeneity, analysis was performed among subgroups‐based type of substance.

## 3 RESULTS

Thirty‐three studies met the inclusion criteria with 51,001 male adolescents aged 16.37 ± 1.68. In Figure 1, the flowchart of extraction and selection of articles was illustrated. It is noticeable that other studies acquire the minimum qualifications required to enter the study.

**Figure 2:**
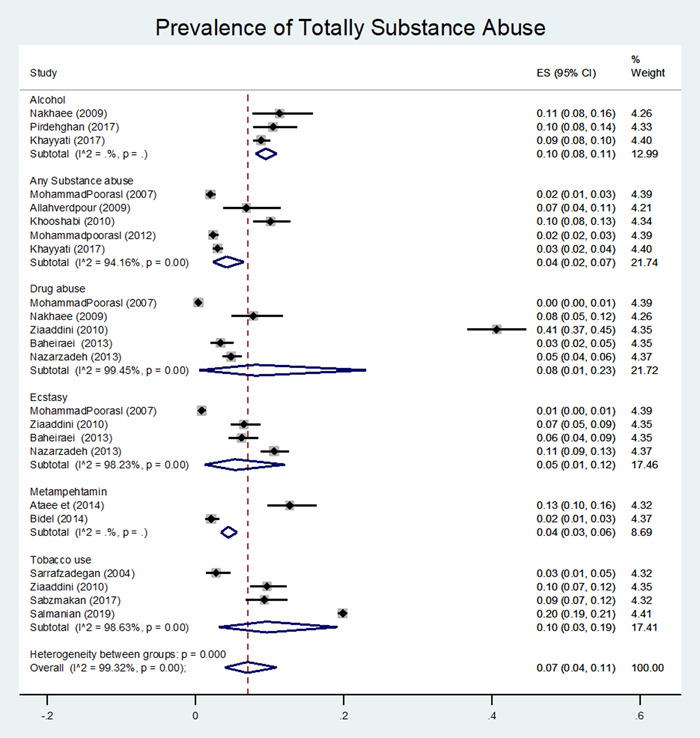
Forest plot of the pooled prevalence of total substance abuse in Iranian male adolescents by the type of substance

Results integration of 15 studies estimated the prevalence of substance use among male adolescents. Overall prevalence was 7% (4%, 11%). I2 index reported 99% indicating high heterogeneity among studies. Analysis among subgroups depending on the type of substances showed that the overall prevalence of alcohol consumption 10% (8%–11%), and use of any addictive substances 4% (2%–7%) were estimated respectively. In addition, ecstasy pill 5% (1%–12%), methamphetamine 4% (3%–6%), and tobacco use 10% (3%–19%, Figure 2) were reported.

**Figure 3:**
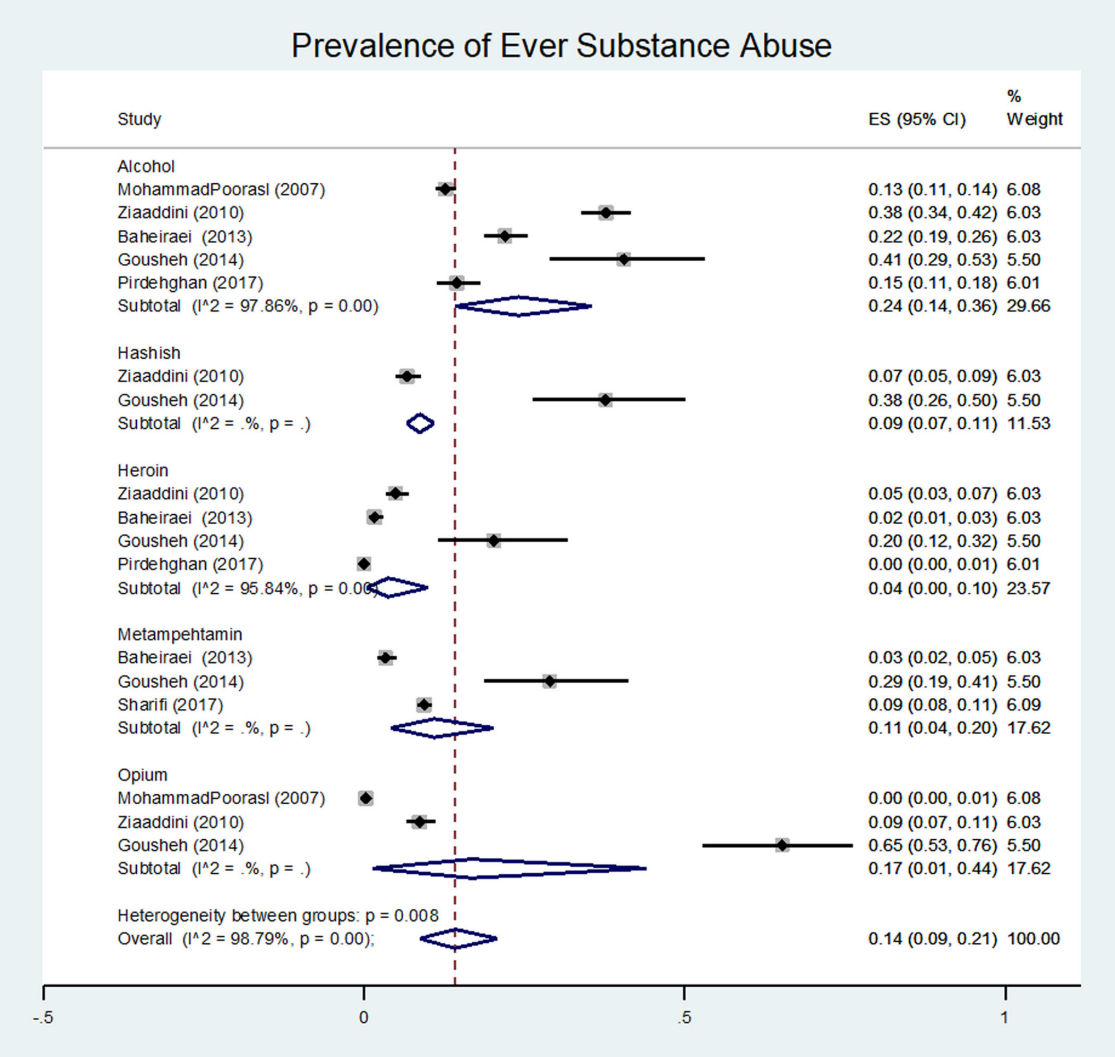
Forest plot of the pooled prevalence of ever substance abuse in Iranian male adolescents by the type of substance

Figure 3 illustrated a combination of the results of six studies reporting the prevalence of at least one use (one of the addictive substances) among male adolescents relying on substance type. Similarly, prevalence of alcohol consumption was 24% (14%–36%), cannabis 9% (7%–11%), heroin 4% (0%–10%), amphetamine 11% (4%–20%), methamphetamine 4% (3%–6%) and the prevalence of opium use 17% (1%, 44%, Figure 2) were estimated respectively.

Figure 3 likewise the other previous figure depicted a combination of the various studies estimating the prevalence of substance use regard to type and frequency of substance use.

## 4 DISCUSSION

This study aimed to investigate the prevalence of substance use among male adolescents in Iran from 2004 to 2020. Eight hundred and five related articles were extracted by the research group. After reaching mentioned criteria, 33 articles were selected to assess.

Overall the prevalence of substance use among Iranian male adult in this study was high. Between all substances opioid, amphetamines and its derivatives and alcohol had more prevalence. It is necessary for health policy makers to pay more attention to these facts.

According to findings of this study being similar to the results of the epidemiological study of Amin Ismaili et al.^1^ 12‐month prevalence of disorder of any illegal substance use was 2.44%. Correspondingly, the prevalence of opium use was estimated at 17%, which is compatible with the findings of Ismaili et al.'s study.^1^ Besides, this study reported opium use as the most common substance use. The results of Akbari's study showed that the highest rate of substance dependence in Medium‐term residential centers for substance use treatment (MTRC) belongs to hard opioids and soft opioids with a prevalence of 43% and 29.5% respectively. Soft opioids include opium (shireh; the condensed extract of remnants of smoked opium), morphine, methadone, and tramadol, and (b) hard opioids including heroin and crystalized form of heroin.^50^

It is supposed that the prevalence of heroin use is 4% on the contrary the results of Moradi's study showed that the prevalence of heroin injection among prisoners in Iran was 6.16%. This difference could be since there are diversity and heterogeneity in the sample of this study. Because of the irritability effect of heroin and high impulsivity upon users, it is presumable that heroin use bumps the number of criminals up remarkably. Becoming Confined population to adolescence in this study, the rate of injection among adolescents in Iran is low. In the same way, the findings of the study by Rahimi Movaghar and colleagues showed the average age of the users who have their first injection among Iranian youth was 25.8 years.

The prevalence of various substances in adolescents depends on geographical, cultural, and traditional conditions. For example, in Iran, the prevalence of opioid use is more common than in other parts of the world. In a systematic review of Substance use among adolescents in sub‐Saharan Africa, the overall prevalence of “any substance use” in sub‐Saharan Africa was 41.6%, with the highest rate in Central Africa at 55.5%. The use of caffeine‐containing products (including coffee or kola nut) was most predominant at 41.2% but limited to West Africa. These were followed by alcohol at 32.8%, tobacco products at 23.5%, khat at 22.0%, and cannabis at 15.9%. Other used substances included depressants at 11.3%, amphetamines at 9.4%, heroin at 4.0%, and cocaine at 3.9%.^51^

## 5 CONCLUSION

The prevalence of substance use among male adolescents is high, so it is indispensable for researchers to pay special attention to this issue.

## AUTHOR CONTRIBUTIONS

**Jalil Hosseini**: Conceptualization. **Ehsan Shojaeefar**: Conceptualization. **Parham Pooladgar**: Writing – original draft. **Fereshteh Aliakbari**: Writing – original draft. **Mostafa Hamdieh**: Writing – review & editing. **Ali Kheradmand**: Writing – review & editing. **Maryam Ganji**: Writing – review & editing. **Mahta Abbasi Fashami**: Writing – original draft.

## CONFLICT OF INTEREST

The authors declare no conflict of interest.

## TRANSPARENCY STATEMENT

The lead author Mostafa Hamdieh, Ali Kheradmand affirms that this manuscript is an honest, accurate, and transparent account of the study being reported; that no important aspects of the study have been omitted; and that any discrepancies from the study as planned (and, if relevant, registered) have been explained.

## Data Availability

The data that support the findings of this study are available on request from the corresponding author. The data are not publicly available due to privacy or ethical restrictions.
